# Repetitive transcranial magnetic stimulation of the cerebellum improves ataxia and cerebello-fronto plasticity in multiple system atrophy: a randomized, double-blind, sham-controlled and TMS-EEG study

**DOI:** 10.18632/aging.103946

**Published:** 2020-10-21

**Authors:** Penghui Song, Siran Li, Suobin Wang, Hua Wei, Hua Lin, Yuping Wang

**Affiliations:** 1 Department of Neurology, Xuanwu Hospital, Capital Medical University, Beijing 100053, China; 2 Central Laboratory, Xuanwu Hospital, Capital Medical University, Beijing 100053, China; 3 Beijing Geriatric Medical Research Center, Beijing 100053, China; 4 Beijing Key Laboratory of Neuromodulation, Beijing 100053, China

**Keywords:** multiple system atrophy, ataxia, cerebellar cortex, iTBS, TMS-EEG

## Abstract

Cerebellar ataxia is the predominant motor feature of multiple system atrophy cerebellar subtype (MSA-C). Although repetitive transcranial magnetic stimulation (TMS) of the cerebellum is growingly applied in MSA, the mechanism is unknown. We examined dynamic connectivity changes of 20 patients with MSA and 25 healthy controls using TMS combined with electroencephalography. Observations that significantly decreased dynamic cerebello-frontal connectivity in patients have inspired attempts to modulate cerebellar connectivity in order to benefit MSA. We further explore the therapeutic potential of a 10-day treatment of cerebellar intermittent theta burst stimulation (iTBS) in MSA by a randomized, double-blind, sham-controlled trial. The functional reorganization of cerebellar networks was investigated after the end of treatment in active and sham groups. The severity of the symptoms was evaluated using the Scale for Assessment and Rating of Ataxia scores. Patients treated with active stimulation showed an improvement of cerebello-frontal connectivity and balance functions, as revealed by a significant decrease in the ataxia scores (P < 0.01). Importantly, the neural activity of frontal connectivity from 80 to 100 ms after a single TMS was significantly related to the severity of the disease. Our study provides new proof that cerebellar iTBS improves motor imbalance in MSA by acting on cerebello-cortical plasticity.

## INTRODUCTION

Multiple system atrophy (MSA) is a fatal, progressive, neurodegenerative disease associated with disability and poor quality of life that ultimately results in an immobilized, bedridden patient. MSA with predominant cerebellar dysfunction (MSA-C) typically presents with cerebellar ataxia, and therapeutic options remain limited. There is an urgent attempt to develop new therapeutic strategies to slow the progression of MSA-C. Repetitive transcranial magnetic stimulation (rTMS), a non-invasive brain stimulation technique, influences neuroplasticity in the short and long term, both at the local cortex under the stimulating coil and at the network level throughout the brain [1]. rTMS has been approved by the Food and Drug Administration to treat depression. Recent studies have highlighted its treatment potential for other brain diseases [2].

Target selection for rTMS has considerable impact on its therapeutic effect. The left primary motor area (M1) [3, 4], cerebellum, and bilateral M1 have been studied as potential treatment targets in patients with MSA [5]. Brain imaging of MSA showed cerebellar volume atrophy and hypometabolism. Cerebellum dysfunction contributes substantially to motor imbalance and disability [6, 7]. Based on numerous cerebello-cerebral networks and the high responsiveness of the cerebellar cortex to magnetic stimuli [8], the cerebellum is an ideal target for modulation of the dysfunction of the cerebellar circuitry in MSA-C. Although rTMS neuromodulation is promising, little is known about the impact of rTMS on brain function networks in MSA.

There is an urgent need to integrate clinical behavioral characteristics with data from electrophysiological or functional imaging techniques. As poor temporal resolution and indirect measure of neural activity by the BOLD signal, functional magnetic imaging (fMRI) is not the best method to detect processing in neural networks. Transcranial magnetic stimulation combined with electroencephalography (TMS-EEG) enables direct probing of cortical reactivity and assessment of cortico-cortical connections on a millisecond time-scale. Most importantly, TMS-EEG enables us to study the cortical response to the TMS pulse and to characterize brain functional connectivity in time, spatial, and frequency domains [9]. Hence, the TMS-EEG approach provides new insights into measuring cortical connectivity and plasticity in cortical circuits following neuromodulatory brain stimulation.

To our knowledge, there have been no studies applying TMS-EEG measures to investigate dynamic cerebello-fronto connectivity in patients with MSA and healthy subjects. Elucidating these differences is key to understanding both the neurophysiological mechanisms of cerebellum disorder and treatment effects [10]. We hypothesized that intermittent theta burst stimulation (iTBS), excitatory rTMS of the cerebellum, might improve motor symptoms in patients with MSA by increasing the neural connections of fronto-cerebellar circuits. To test this, we took advantage of adaptive directed transfer function (ADTF) analysis [11, 12] of TMS-EEG to investigate the effect of rTMS over the cerebellum on the information flow of underlying fronto-cerebellar networks with the potential to elicit behavioral changes.

## RESULTS

Twenty patients with MSA and 25 healthy controls participated in Experiment 1 (Table 1). Fifty patients with MSA took part in Experiment 2. They were randomly assigned to the real rTMS group or the sham rTMS group (Table 2). All patients completed the entire study without adverse side effects. There were no significant differences in demographic and clinical characteristics of the participants in Experiment 1 and Experiment 2.

**Table 1:** Demographic and clinical characteristics of Experiment 1.

	**MSA**	**HC**	**p-value**
N	20	25	-
Age, years	51.1 ± 9.2	51.9 ± 10.3	n.s
Gender, % female	50	44	n.s
Age at onset, years	49.4 ± 8.3	-	n.s
Disease duration, years	2.3 ± 0.7	-	-

MSA: multiple system atrophy, HC: healthy controls.

**Table 2:** Demographic and clinical characteristics of Experiment 2.

	**Real rTMS**	**Sham rTMS**	**p-value**
N	25	25	n.s
Age, years	53.1 ± 8.1	53.2 ± 9.4	n.s
Gender, % female	44	40	n.s
Age at onset, years	50.9 ± 7.7	48.7 ± 14.1	n.s
Disease duration, years	2.7 ± 1.1	2.5 ± 0.9	n.s

rTMS: repetitive transcranial magnetic stimulation.

### Differences in the time-varying EEG network patterns between healthy controls and MSA patients

**Figure 1:**
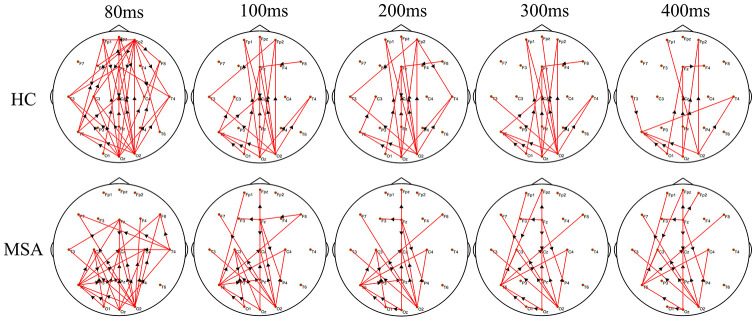
**The difference in the time-varying EEG network patterns between HCs and MSA patients.** Time (millisecond): after single-pulse TMS. Red lines: enhanced connections; black arrows: the direction of information flow; MSA: multiple system atrophy; HC: healthy control.

**Figure 2:**
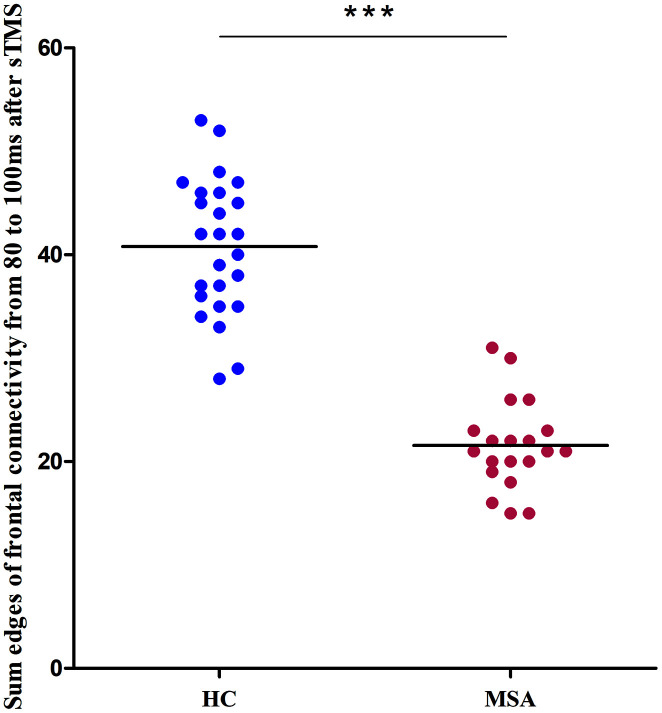
**Differences between healthy controls (blue) and MSA patients (red) in the sum of the edges of frontal connectivity from 80 to 100 ms after sTMS in Experiment 1.** ***P<0.001. sTMS: single-pulse transcranial magnetic stimulation.

We recruited 20 MSA patients (mean SARA score= 20, SD=5.2) and 25 healthy controls (mean SARA score=0.6, SD=0.7). Figure 1 shows the differences in the time-varying EEG network patterns between controls and patients. The connections in the frontal area were insufficient in the MSA patients compared with healthy controls, particularly at 80ms. We calculated the sum of the edges of frontal connectivity from 80 to 100 ms after single TMS in patients and controls. It showed that the number of frontal connecting edges of MSA was significantly less than that of healthy controls (Figure 2).

### Clinical outcomes of iTBS treatment

**Figure 3:**
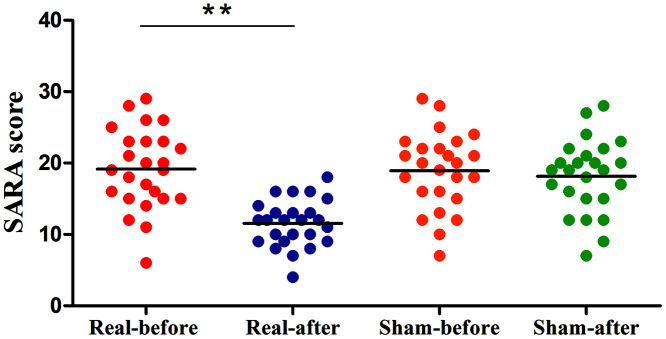
**Differences in SARA scores of the pre- and post-treatment in the real rTMS and sham group separately.** The real rTMS group exhibits decreases in SARA scores after treatment compared with the sham rTMS group. **P<0.01. SARA: scale for the assessment and rating of ataxia.

The clinical data of Experiment 2 is shown in Figure 3. After a total of 10 days of real treatment, most of the patients experienced improvement in ataxia. None of the patients worsened. The SARA scores of the real rTMS group declined significantly after treatment compared to baseline (pre vs. post, 19.0±5.67 vs. 11.5±3.26). The more serious the patients’ataxia symptoms were before treatment, the more the SARA scores improved after the real rTMS treatment. In contrast, the SARA scores of the sham group were unchanged (pre vs. post, 17.7±6.0 vs. 17.3±6.0).

### Differences in the time-varying EEG network patterns between the real and the sham therapy

**Figure 4:**
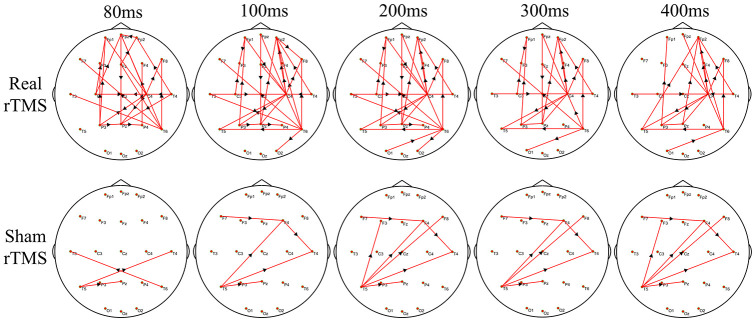
**The difference in time-varying EEG network patterns between the real rTMS group and the sham group after therapy.** Time (millisecond): after single-pulse TMS. Red lines: enhanced connections; black arrows: the direction of information flow; rTMS: repetitive transcranial magnetic stimulation.

**Figure 5:**
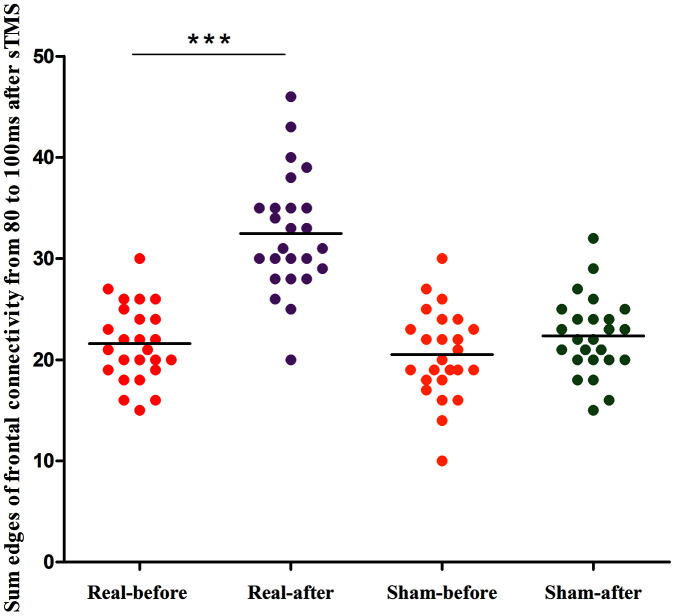
**Differences in the edges of frontal connectivity from 80 to 100 ms of the pre- and post-treatment in the real rTMS and sham group separately.** ***P<0.001. sTMS: single-pulse transcranial magnetic stimulation.

Figure 4 shows the group differences of the brain connection in the corresponding time-varying EEG network patterns of MSA patients after the real and sham stimulations. Frontal connectivity of post-treatment increased significantly than that of pre-treatment in the real rTMS group, but no change in the sham group. We calculated the sum of the edges of frontal connectivity from 80 to 100 ms after single TMS before and after the real or sham stimulations. The number of frontal connecting edges of the real rTMS group was significantly increased in comparison to the sham rTMS group after 10 days of therapy (Figure 5).

### Correlation between frontal connectivity and SARA score

**Figure 6:**
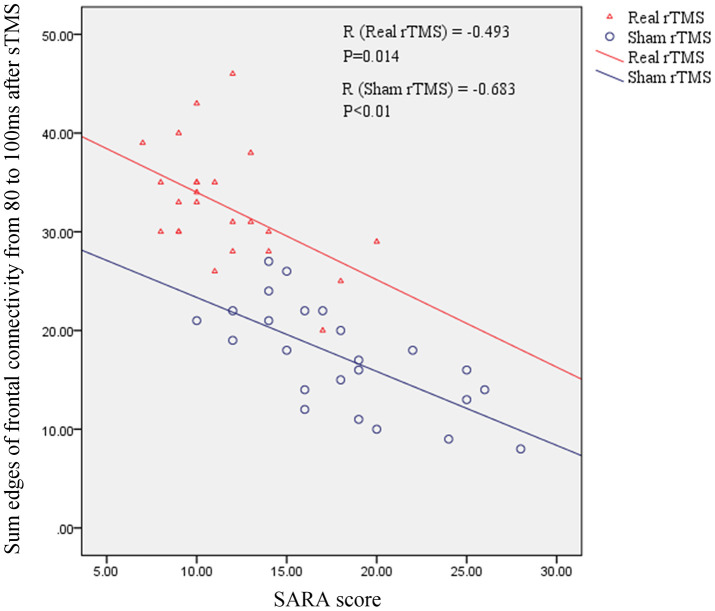
**The relationship between the sum of the edges of frontal connectivity from 80 to 100 ms and the SARA score in patients with MSA.** There is a significant negative correlation between the SARA score and the sum of the edges of frontal connections from 80 to 100 ms after rTMS intervention, independent of real or sham rTMS. SARA: scale for the assessment and rating of ataxia; rTMS: repetitive transcranial magnetic stimulation; sTMS: single-pulse transcranial magnetic stimulation.

To better assess the association between frontal connectivity and we calculated the sum of the edges of frontal connectivity from 80 to 100 ms after single TMS in patients with MSA after iTBS intervention. The number of the edges was negatively correlated with the SARA scores in the two groups (Figure 6). These findings indicate that the edges of the frontal connectivity may be related to the severity of the MSA disease.

## DISCUSSION

This study examines differences in cerebello-frontal connectivity in MSA patients and healthy controls using TMS-EEG measures. We found that cerebellar iTBS has a significant beneficial effect on motor imbalance in patients with MSA-C, as demonstrated by the decrease in SARA scores. Importantly, these changes are modulated by an enhancement in neural activity in the cerebellar-frontal network as measured by TMS-EEG.

Compared to healthy controls, MSA patients showed significantly decreased dynamic information processing at the frontal cortex during single TMS over the cerebellum (especially prefrontal connectivity) mainly within the time window of 80 ms. These results provide the first evidence of reduced electrical activity at prefrontal cortical areas by cerebellar-evoked activity in MSA patients. Although the interconnection between the cerebellum and prefrontal cortices has been well-established in humans using fMRI [16–18] or neurophysiology methods [19, 20], very little is known about accurate dynamic information flow change after the TMS pulse. Koch et al. [21] revealed an increase in neural activity from 60 to 90 ms following a single-pulse TMS applied over the precuneus using TMS-evoked potentials and global mean field power amplitude. We employed ADTF, based on the time-varying outflow of information at exact time series and space distribution, to more precisely measure brain network activity. Our results suggest that neural dysfunction between the cerebellum and prefrontal cortices occurs mostly in the early phase of the evocation. Therefore, modulation of the disrupted cerebello-frontal connectivity in the early stages is a key intervention strategy.

Previous research has demonstrated temporary functional improvement after sessions of high frequency cerebellar rTMS in patients with ataxia [22–25] and the enhancement of cerebellar ataxia after anodal cerebellar tDCS has also been reported as a long-term clinical effect [26–28]. In the present work, a 10-day treatment with cerebellar iTBS showed significant differences between pre- and post- real rTMS treatment. Conversely, no effects were found between pre- and post- sham rTMS. We found that 10 days of cerebellar iTBS treatment enhanced the prefrontal connectivity at 80-100 ms and 200-400 ms in MSA patients. Additionally, clinical SARA scores were significantly improved in MSA patients after active vs. sham rTMS treatment, which is in agreement with previous findings [3, 4, 8]. This motor improvement was accompanied by an enhancement of brain connectivity, consistent with the neurophysiological features of cerebellum disconnection in MSA. A longer therapy duration is required in clinical practice for chronic degeneration disorder [21] or serious motor deficits [20]. Manor et al. [23] provided preliminary evidence that 4-week rTMS targeting the cerebellum greatly improve standing postural control for at least 1 month in patients with spinocerebellar ataxia. Together, these findings provide evidence that non-invasive neuromodulation of network dysfunction, through stimulation of the cerebellum, may be an effective strategy to ameliorate motor imbalance and disability in patients with ataxia. Although our sample size was relatively small, our results provide the basis for a larger, multicenter clinical trial in the future aimed at evaluation of the beneficial effects of cerebellar iTBS in slowing motor disability in MSA when applied for a longer period of time (i.e., six months).

Neurobiologically, rTMS produces an LTP-like effect and facilitates synaptic plasticity in animal models [29, 30]. Since TBS mimics neuron oscillatory rhythms [31, 32], it is reasonable to apply iTBS to the human brain cortex using TMS [31]. In particular, iTBS strongly activates the neural informational flow of the motor cortex and cerebellum [33]. Recently, a randomized clinical trial found that the gait and balance functions in patients with hemiparetic stroke improved after a 3-week course of cerebellar iTBS. Further studies into this mechanism revealed that these changes were paralleled by activation of the cerebello-cortical plasticity, as measured by TMS-EEG. Motor improvement in MSA patients was associated with cerebellar activation after 5Hz rTMS over M1, as identified by fMRI [4]. Using virus transneuronal tracers, animal studies have demonstrated that cerebellar output reaches the frontal cortex [34]. Overall, the cerebellar-frontal circuits are critical for motor coordination. The quantity and activity of functional units provide the basis for information storage in cerebellar-frontal connectivity at adulthood [32]. We employed TMS-EEG to investigate cerebellar-evoked electrical activity in frontal cortical areas in MSA-C patients. ADTF analysis was used to construct the time-varying networks and assess the dynamic information processing during TMS disturbance [35]. Reduced functional storage was observed in multiple system atrophy patients, especially in the early 80 ms. Motor imbalance was improved by increasing functional connectivity after cerebellar iTBS treatment. Our results highlight the importance of functional connectivity storage in the early 80 ms, which may be a potential neurophysiological marker for predicting MSA severity.

There are some limitations in the study. First, our result is limited by the relatively small sample size. There is a great need to plan large scale, sham-controlled trials to confirm the benefits of neuromodulation of the cerebellum. Second, a cross-over design might be better in a second set. Due to limited time and funding, we did not do this in this study. In addition, the potential artifacts were mainly within 60 ms after delivering the TMS pulse. The data from 50 to 70 ms were not analyzed, although important information is likely included in that range. Finally, the relatively small number of recording electrodes made precise spatial definition difficult.

## CONCLUSION

In conclusion, our findings provide novel evidence that iTBS over the cerebellum is potentially an effective strategy to improve motor symptoms by enhancing cerebellar-frontal connectivity reorganization in patients with MSA.

## MATERIALS AND METHODS

### Experimental procedure

We conducted two consecutive experiments. All of the enrolled participants in two experiments completed baseline assessments and a structural brain MRI. Experiment 1 was designed to clarify dynamic brain connectivity changes induced by single-pulse TMS in the time-varying network in healthy controls and patients with MSA. Participants were asked to relax and stay positioned on a semi-reclined chair for 20 minutes when recording the TMS-EEG data.

**Figure 7:**
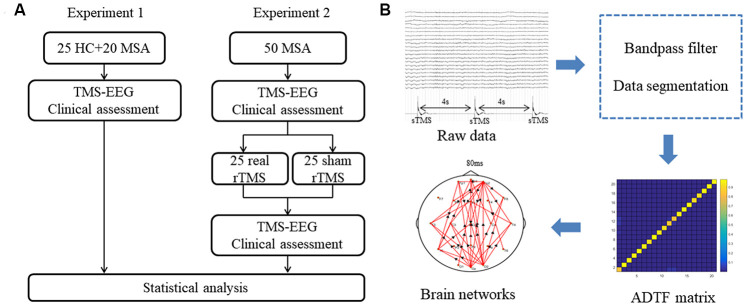
(**A**) Schematic representation of experimental design. (**B**) Analysis procedure for TMS-EEG data. MSA: multiple system atrophy; HC: healthy control; TMS-EEG: transcranial magnetic stimulation–electroencephalogram; rTMS: repetitive transcranial magnetic stimulation; sTMS: single-pulse transcranial magnetic stimulation; ADTF: adapted directed transfer function.

Experiment 2 was a randomized, double-blind, sham-controlled study. The objective is to examine the therapeutic effects of iTBS applied over the cerebellum in MSA patients and to observe changes in the time-varying network. In the second experiment, all patients firstly underwent 20 minutes of TMS-EEG recording. Their limb kinetic function was evaluated using the scale for the assessment and rating of ataxia (SARA). Then, patients were randomly assigned to receive 10 days of real or sham rTMS therapy. At the end of the therapy day, 20 minutes of TMS-EEG recording and SARA test was again made. (Figure 7A). The primary observation index was functional connectivity in the cerebello-fronto network using the TMS-EEG technique. The SARA score was the secondary index. All researchers conducting clinical assessments were blinded to the experimental groups.

### Participants

The patients were recruited between March 2017 and October 2019 from the Department of Neurology, Xuanwu Hospital, Capital Medical University. Inclusion criteria for patients with MSA were as follows: (1) Met the clinical criteria for probable MSA-C [13]. (2) Age 18–75. (3) No other neurodegenerative diseases. Exclusion criteria were as follows: (1) Unstable neurological illness or concomitant medical condition. (2) Clinically significant abnormalities on screening (e.g., basic lab work or ECG abnormalities.). (3) Concurrent participation in another clinical study, history of substance abuse, psychiatric illness, legal incapacity or limited legal capacity. (4) Metal in the head, history of neurosurgical procedures, ferromagnetic bioimplants, metallic paint, history of seizure disorder, claustrophobia, current usage of bupropion or other medications that may increase the risk of TMS-induced seizures. (5) A positive pregnancy test. This study was approved by the Ethics Committee of Capital Medical University Xuanwu Hospital. Every subject provided written informed consent to participate in this study.

### Measurements of rest motor threshold

Single-pulse TMS was applied with a figure-of-eight coil (70 mm diameter) connected to a monophasic Magstim stimulator (Magstim Company Ltd., London, UK). The resting motor threshold (RMT) was defined as the lowest stimulation intensity that could produce at least five motor evoked potentials with wave amplitudes >50 mV among 10 trials in the right first dorsal interosseous muscle. The surface electromyography was recorded using disc-shaped Ag-Cl electrodes that were placed in a tendon-belly arrangement. The stimulating coil was positioned tangentially to the skull with the coil handle pointing backward and laterally at 45°from the anterior-posterior axis.

### TMS-EEG data acquisition

TMS-EEG data (20 min) were acquired using a magnetic field-compatible EEG amplifier (Yunshen Ltd, Beijing, China). The cap (Greentek Ltd, Wuhan, China) with 32 TMS-compatible electrodes were positioned according to the 10-20 montage. The sample rate was 1024 Hz. The electrode impedances were maintained below 5 kΩ. The AFz channel was the reference and nasal tip electrodes served as ground. Single TMS (sTMS) was stimulated in the right cerebellum [14] (1 cm inferior and 3 cm right to the inion) at 80% RMT. Each sTMS was applied at an interval of 4s to avoid a TMS effect. Synchronous EEG recordings were made. Subjects wore earplugs to shield environmental noise and coil discharge noise.

### rTMS stimulation treatment parameters

In the second experiment, rTMS was delivered using a Magstim Rapid 2 stimulator (Magstim, Co. Ltd, UK). iTBS therapy applied over the bilateral cerebellum using the same scalp coordinates (1 cm inferior and 3 cm left/right to the inion). One iTBS session consisted of the following parameters: a burst of 3 pulses at 50 Hz repeated at 200-ms intervals, a short train lasting 2 seconds, an inter-train interval of 8 seconds, 300 pulses in a session. The stimulation intensity for the iTBS was set at 80% of the RMT. Three iTBS sessions were performed separately on the left and right cerebellum with a 5-min interval between sessions (1800 pulses in total). The sham stimulation was performed using a Magstim placebo coil, which mimics the typical “click” of the genuine coil without magnetic stimulation. The procedures and parameters of iTBS sham stimulation were the same as the real stimulation. The treatment was applied once a day for two weeks with rest on the weekends (total of ten days rTMS therapy).

### EEG data analysis and statistical

The pre-processing and time-varying network analyses were performed on the MATLAB platform (R2015b, The Mathworks, USA). The pre-processing of EEG data included a bandpass filter (3-30Hz) and data segmentation. We used single TMS disturbance as the stimulus labels. For every disturbance event label, the time point corresponding to the peak of the label was set as time ‘‘0’’. Data corresponding to 0.5 s before (as baseline) and 0.4 s after ‘‘0’’ were extracted. Each segment length was 0.9 s. Considering the artifacts produced by the TMS on the EEG signals, we focused on the changes in brain networks at 80-400 ms after the single TMS stimulation. To capture the characteristics of brain connections, time-varying network analysis requires several steps to build a reliable network. First, in order to reduce the calculation load, the sampling rate is 128Hz in the final analysis by 8 times down-sampling during data processing. Next, we built a time-varying multivariate adaptive autoregressive (tv-MVAAR) model and calculated the ADTF matrix. This procedure is available in the supplementary material appendix. Last, we mapped the brain networks as previously described [12, 15] (Figure 7B).

Considering that phase randomization preserves the spectral structure of the time series, Fourier coefficient phases were randomly and independently shuffled to produce the corresponding reference signals. This procedure was repeated 300 times for each segment of each subject. An empirical distribution of ADTF values was created under the null hypothesis of no causal interaction in each edge. Then, the time-varying networks, which corresponded to each single trial, were further averaged across all the artifact-free trials. This averaging procedure gave the final time-varying networks.

The time-varying EEG networks were analyzed using two-tailed t-test. Matlab was utilized to identify the dynamic network patterns before and after the iTBS treatment. The means and variances were used to calculate the Gauss cumulative distribution. The dynamic networks were calculated with a significance of 0.05.

### Statistical analysis

Clinical assessment data were analyzed using SPSS software version 19.0 (IBM Corp., Armonk, NY, USA). Demographic and clinical variables were compared using between-group two-sample, two-tailed t-tests or chi-squares. The sum of the edges of frontal connectivity from 80 to 100 ms after single TMS were compared using between-group two-sample, two-tailed t-tests. Pearson's correlation test was performed to examine the correlation between the sum of the edges of frontal connectivity from 80 to 100 ms and the SARA scores.

## Supplementary Material

Supplementary Materials
