# Supplementary material for: Repetitive transcranial magnetic stimulation of the cerebellum improves ataxia and cerebello-fronto plasticity in multiple system atrophy: a randomized, double-blind, sham-controlled and TMS-EEG study
Source: Aging (Albany NY). 2020 Oct 21;12(20):20611–22. doi: 10.18632/aging.103946 (PMC7655163; doi:10.18632/aging.103946)
Supplement: Supplementary Materials [file aging-12-103946-s001..pdf]

SUPPLEMENTARY MATERIALS

The process of build time-varying multivariate adaptive autoregressive (tv-MVAAR) model and calculate ADTF matrix.

Time-Varying Multivariate Adaptive Autoregressive (tv-MVAAR) Model

For each artifact-free segment, the tv-MVAAR model was defined as

$$X(t) = \sum_{i=1}^{p} A(i,t)X(t-1) + E(t) \qquad (1)$$

where X(t) represents the data vector of the EEG signal, E(t) represents the multivariate independent white noise, and X(i,t) represents the matrix of tv-MVAAR model coefficients that are estimated by the Kalman filter algorithm. Represents the order of the model that is automatically determined by the Akaike Information Criterion (AIC) within the range of 2–20 as,

$$AIC(p) = \ln\left[\det(\chi)\right] + 2M^2 p / N \qquad (2)$$

where M represents the number of the electrodes, p represents the optimal order of the model, N represents the number of the time points of each time series and χ represents the corresponding covariance matrix.

Adaptive Directed Transfer Function Parameters A(f,t) and H(f,t) in the frequency domain are defined as follows;

$$A(f,t) = \sum_{k=0}^{p} A_k(t)e^{-j2\pi f \vartriangle tk} \qquad (3)$$

$$A(f,t)X(f,t) = E(f,t) \qquad (4)$$

$$X(f,t) = A^{-1}(f,t)E(f,t) = H(f,t)E(f,t) \qquad (5)$$

where Ak denotes the matrix of the tv-MVAAR model coefficients, X(f,t) and E(f,t) are the Fourier transformations of X(t) and X(t) represents the frequency domain, respectively.

Moreover, the normalized ADTF describing the directed flow from the jth to the ith node is defined by Equation (6), and the final integrated ADTF is defined in Equation (7) within the frequency band of interest as follows;

$$\gamma_{ij}^2(f,t) = \frac{\left|H_{ij}(f,t)\right|^2}{\sum_{m=1}^{n}\left|H_{im}(f,t)\right|^2} \qquad (6)$$

$$Q_{ij}^2(t) = \frac{\sum_{k=f_1}^{f_2} \gamma_{ij}^2(k,t)}{f_2 - f_1} \qquad (7)$$

The normalized total information outflow of the jth node is further estimated in Equation (8) as;

$$Q_j^2(t) = \frac{\sum_{k=1}^{n} Q_{kj}^2(t)}{n-1}, \; for \; k \neq j \qquad (8)$$

where n is the total number of nodes. When each node (n) has been calculated for each sample time point (t), a directional edge (i to j) can be displayed. From Equation 8, we can derive an outflow that denotes the time-varying of each node across different time points.
